# Overflow urinary incontinence as an early manifestation of neuronal intranuclear inclusion disease (NIID): a case report

**DOI:** 10.1186/s13256-026-06049-0

**Published:** 2026-04-21

**Authors:** Ming Yang, Zhaoping Wu, Guoyuan Ju, Tingting Duan

**Affiliations:** Department of Neurology, The Quzhou Affiliated Hospital of Wenzhou Medical University, Quzhou People’s Hospital, Quzhou, Zhejiang China

**Keywords:** Case report, Magnetic resonance imaging, Neuronal intranuclear inclusion disease, NOTCH2NLC, Urinary incontinence

## Abstract

**Background:**

Neuronal intranuclear inclusion disease (NIID) is a rare neurodegenerative disorder with highly heterogeneous clinical manifestations, often leading to misdiagnosis. Urinary incontinence as the initial and predominant symptom of NIID has rarely been reported.

**Case presentation:**

A Chinese male in his early seventies presented with an 8-year history of nocturnal urinary incontinence and a 4-year history of progressive memory loss. Detailed clinical, neuroimaging, electrophysiological, and genetic assessments were performed. Magnetic resonance imaging (MRI) showed high-intensity signals along the corticomedullary junction on diffusion-weighted imaging (DWI). Electrophysiological studies indicated peripheral neuropathy in all four limbs. Genetic analysis via polymerase chain reaction (PCR) combined with capillary electrophoresis identified a GGC repeat expansion (115 repeats) in the 5′ untranslated region (5′ UTR) of the NOTCH2NLC gene, confirming the diagnosis of NOTCH2NLC-associated NIID.

**Conclusion:**

This case highlights the diagnostic challenges of NIID presenting primarily with overflow urinary incontinence. It emphasizes the importance of considering NIID in patients with unexplained urinary dysfunction accompanied by cognitive decline, and confirms that early genetic testing is essential for accurate diagnosis and differential identification from other neurodegenerative diseases.

## Background

Neuronal intranuclear inclusion disease (NIID) is a rare, progressive neurodegenerative disorder that may occur sporadically or follow an autosomal dominant inheritance pattern. Its pathological hallmark is the widespread presence of eosinophilic intranuclear inclusions within the central nervous system (CNS), peripheral nerves, skin, and various visceral organs [1, 2]. Involvement of the CNS may result in cognitive decline, parkinsonism, episodic encephalopathy, and mood disturbances [3], whereas peripheral nerve involvement can cause muscle weakness and sensory abnormalities. Autonomic dysfunction is frequently observed, leading to urinary frequency, retention, or incontinence, as well as orthostatic hypotension and miosis [4].

Previous studies have identified eosinophilic intranuclear inclusions in renal and bladder tissues of NIID patients, with more than half exhibiting urinary dysfunction [5, 6]. Owing to the marked clinical heterogeneity of NIID—particularly in cases dominated by autonomic symptoms—misdiagnosis or delayed diagnosis remains common [2].

In recent years, advances in neuroimaging, skin biopsy, and molecular genetic testing have markedly improved diagnostic accuracy, suggesting that the actual prevalence of NIID may be higher than previously estimated. Magnetic resonance imaging (MRI) typically demonstrates a characteristic curvilinear high-intensity signal along the corticomedullary junction on diffusion-weighted imaging (DWI), which serves as a reliable radiological marker of adult-onset NIID [1, 5, 7]. In 2019, several independent research groups identified an expanded GGC repeat in the 5′ untranslated region (5′-UTR) of the NOTCH2NLC gene as the key genetic cause of NIID, marking the transition of NIID diagnosis into the molecular genetic era [8–11].

## Case presentation

A Chinese male in his early seventies was admitted due to an eight-year history of nocturnal urinary incontinence and a four-year history of progressive memory decline. Eight years ago, the patient developed overflow incontinence secondary to chronic urinary retention without obvious inducement, manifested as frequent micturition, urgent micturition and nocturnal incontinence during sleep with normal daytime urination, accompanied by sexual dysfunction. He was initially evaluated by the urology department and diagnosed with benign prostatic hyperplasia (BPH), for which transurethral resection of the prostate was performed. However, his urinary symptoms did not improve postoperatively.

Four years before admission, his daughter noticed worsening short-term memory loss, characterized by forgetfulness of the recent events, slowed responses, disorientation during outdoor activities, and one episode of getting lost. There were no parkinsonian gait abnormalities, speech disturbances, or tremors. In November 2024, the patient underwent cranial MRI at a local hospital, which revealed multiple ischemic foci and small lacunar infarctions in the bilateral periventricular, centrum semiovale, and basal ganglia regions, accompanied by leukoaraiosis and age-related brain atrophy. Symmetrical abnormalities were also observed in the gray–white matter junctions of the bilateral frontal lobes, suggestive of a demyelinating process. The patient later presented to the urology clinic of our hospital for persistent urinary incontinence and was subsequently referred to the neurology department for further evaluation. The patient had a 10-year history of hypertension with well-controlled blood pressure (110–140/70–90 mmHg) under regular medication. He denied any substance abuse or family history of similar neurological disorders.

Neurological examination upon admission revealed normal mental status, fluent speech, and appropriate responses. Cranial nerve function was intact. Muscle strength and tone in all extremities were normal. Deep and superficial sensations were preserved, and deep tendon reflexes were normal. Mild dysmetria was observed during the left finger–nose test and heel–knee–shin testing on both sides. No pathological reflexes were elicited. Serial neurological re-evaluations consistently confirmed the absence of parkinsonism, cerebellar gait ataxia, and pyramidal signs. The Mini-Mental State Examination (MMSE) score was 24, and the Montreal Cognitive Assessment (MoCA) score was 16 (adjusted for education level). Orthostatic testing showed no significant drop in blood pressure or heart rate.

**Fig. 1:**
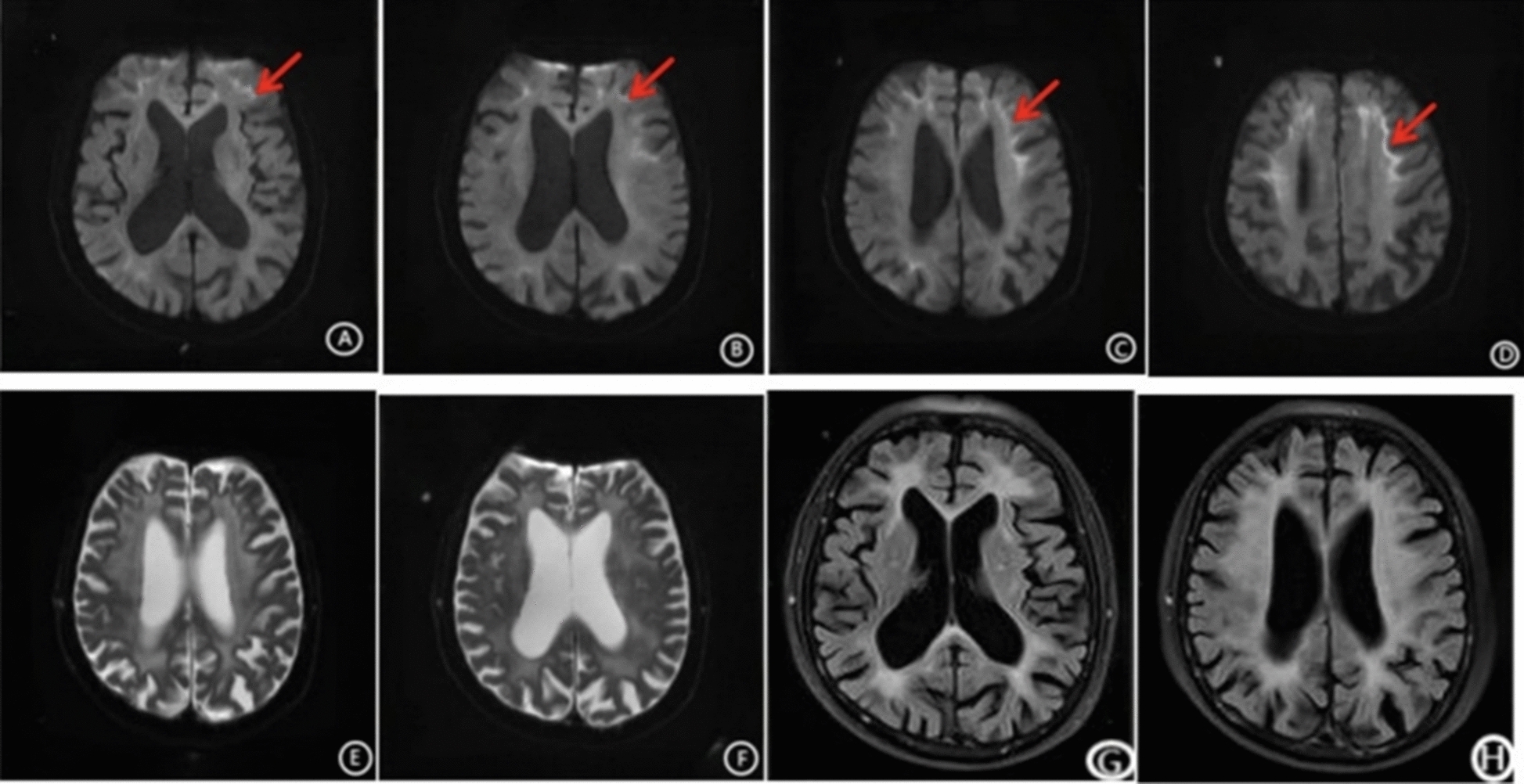
The patient's MRI scanning revealed the following findings: A-D: DWI showed bilateral “ribbon-like” hyperintensities at the corticomedullary junction of the frontal and parietal lobes, resembling the “lace sign”(arrows); E, F: T2-weighted imaging demonstrated bilateral periventricular white matter hyperintensities; G, H: T2 FLAIR sequences revealed diffuse confluent white matter changes

Laboratory and electrophysiological investigations were largely unremarkable, including complete blood count, metabolic panel, vitamin B12, folate, homocysteine, autoimmune, rheumatologic, coagulation, and tumor marker profiles. The patient had subclinical mixed sensorimotor axonal peripheral neuropathy involving all four limbs. Electrophysiologically, the amplitudes of compound muscle action potentials (CMAP) and sensory nerve action potentials (SNAP) were reduced or undetectable in most nerves, with mild slowing of motor conduction velocity (MCV) and sensory conduction velocity (SCV) in the lower limbs and normal F-waves; SNAP was absent in the left ulnar, bilateral median, superficial peroneal and sural nerves. Clinically, no motor or sensory deficits were found (limb muscle strength grade 5, normal deep and shallow sensation), with only mild cerebellar ataxia present, which is consistent with the high prevalence of subclinical peripheral neuropathy in NIID. Neuroimaging and other diagnostic findings: Contrast-enhanced CT of the urinary tract demonstrated bladder wall thickening with multiple diverticula, bilateral hydronephrosis, and an enlarged prostate with calcifications, suggestive of a neurogenic bladder. Bladder ultrasound revealed a significant post-void residual urine volume of 375 mL. Urodynamic testing was not performed due to the lack of specialized institutional equipment. Brain MRI with diffusion-weighted imaging (DWI) showed diffuse high-intensity signals along the corticomedullary junction of the bilateral frontal, temporal, parietal, and occipital lobes, as well as brain atrophy (Fig. 1). Electroencephalography revealed no significant abnormalities. Sleep monitoring excluded sleep apnea but indicated mild nocturnal hypoxemia.

**Fig. 2:**
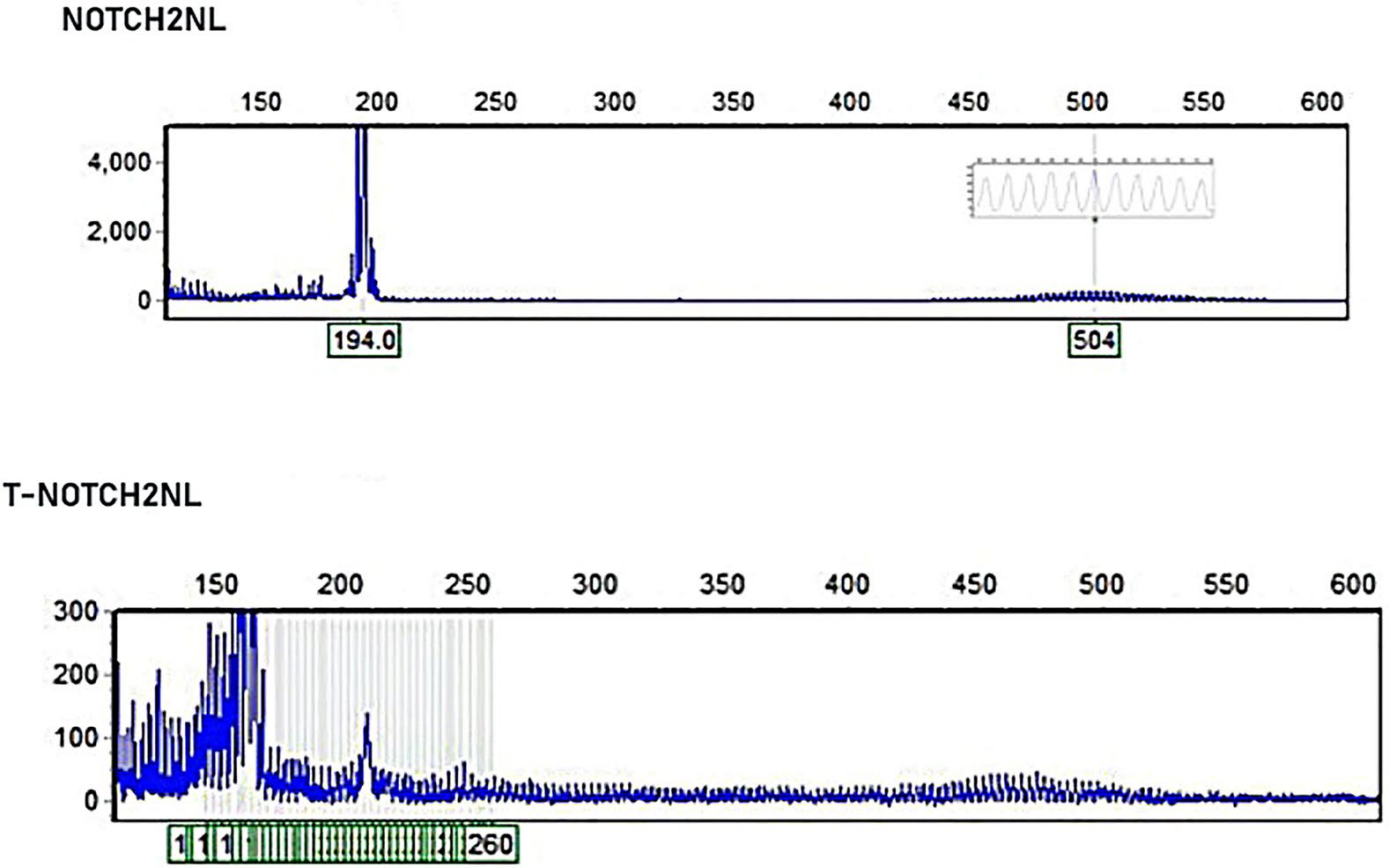
The tested individual exhibited a GGC repeat expansion of 115 in NOTCH2NLC, indicating abnormal amplification, which supported the diagnosis of NIID

Given the characteristic DWI findings—curvilinear hyperintensity along the corticomedullary junction suggestive of NIID—a multidisciplinary case discussion was convened. As skin biopsy was not available at our institution, whole-exome sequencing (WES) was initially performed but yielded negative results. Based on the distinctive clinical and radiological features, repeat genetic testing using PCR combined with capillary electrophoresis was conducted. Repeat-primed PCR (RP-PCR) and amplicon-length PCR (AL-PCR) identified an expanded GGC repeat (115 repeats) in the 5′ untranslated region (5′UTR) of the NOTCH2NLC gene, confirming the diagnosis of NOTCH2NLC-associated NIID (Fig. 2).

**Fig. 3:**
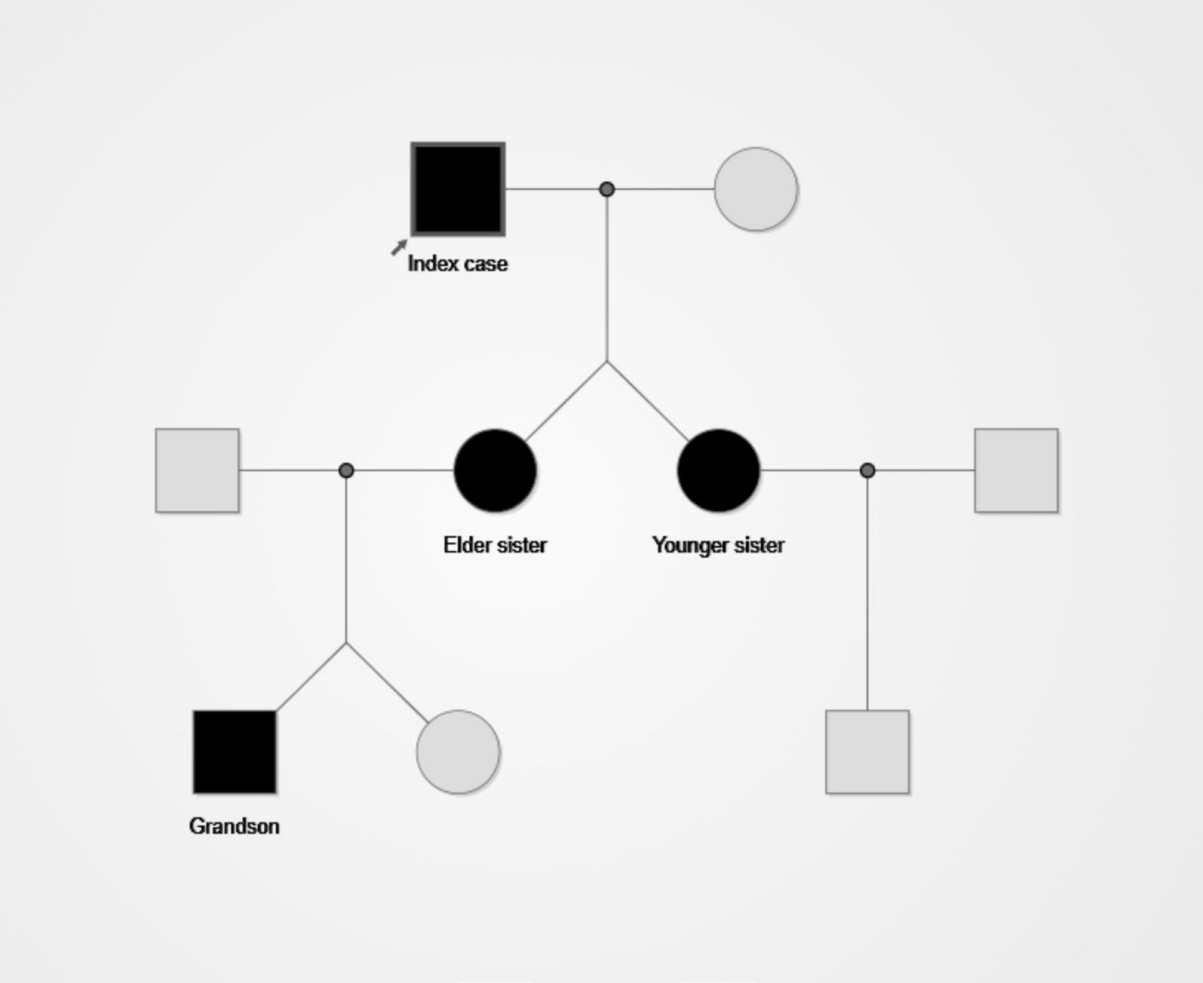
The family pedigree of the patient showed that the patient's two daughters and grandson also carried a GGC repeat expansion mutation in the NOTCH2NLC gene, indicating that this case was a familial hereditary NIID

Subsequent family screening revealed the same GGC repeat expansion in the patient’s two daughters and one grandson, indicating a familial form of NIID (Fig. 3). This finding highlights the genetic characteristics of NIID and the importance of genetic screening for family members, and we have advised the asymptomatic family members carrying this gene mutation to undergo regular clinical surveillance and long-term follow-up for the early detection of disease signs and timely intervention.

During hospitalization, a urinary catheter was inserted due to a post-void residual volume of 375 mL, with 500 mL of urine drained, consistent with severe urinary retention. After discharge, we treated the patient's cognitive impairment and urinary incontinence with donepezil hydrochloride, finasteride tablets, tamsulosin hydrochloride and acupuncture therapy, and conducted regular clinical follow-up. During the follow-up period, no significant improvement in the patient's clinical symptoms was observed.

## Discussion

This case is the first reported familial NIID case characterized by isolated nocturnal overflow incontinence as the sole initial and dominant symptom with an 8-year long course. Although urinary dysfunction is a common manifestation in NIID patients, no prior literature has documented a familial NIID case with such a unique clinical phenotype confirmed by NOTCH2NLC gene detection and family genetic screening. Neuronal Intranuclear Inclusion Disease (NIID) is a rare neurodegenerative disorder primarily caused by GGC repeat expansion in the 5′-untranslated region (UTR) of the NOTCH2NLC gene [12]. Despite approximately 400 cases reported globally by 2022 (predominantly in East Asia) [13, 14], the true incidence of NIID remains unclear due to the lack of systematic epidemiological data and potential underdiagnosis of sporadic cases. Familial NIID, closely linked to NOTCH2NLC GGC repeat expansion [15], typically manifests before 40 years of age, whereas sporadic cases usually onset after 50 [1]. With the widespread application of genetic testing, the diagnostic rate of NIID has significantly improved, accompanied by increasing reports of sporadic cases—suggesting the disease may be more prevalent than previously thought. Notably, our case was initially classified as sporadic due to the absence of a clear family history, with the patient experiencing multiple misdiagnoses across departments before definitive diagnosis via genetic testing. Subsequent familial genetic validation, however, revealed that two daughters and one grandson of the patient also carried the NOTCH2NLC GGC repeat expansion mutation, reclassifying this case as familial NIID. The GGC repeat counts varied among family members: 115 repeats in the proband, 98 and 97 repeats in the two daughters, and 159 repeats in the grandson. Consistent with previous studies demonstrating a correlation between GGC repeat length, disease severity, and age at onset (longer repeats associated with earlier onset and more severe phenotypes) [16, 17]. In this case, the patient developed the disease at the age of 67, while his grandson had a higher number of GGC repeat expansions and may develop an earlier onset and more severe symptoms in the future, and the grandson has not presented with any NIID-related clinical symptoms to date. This finding highlights the critical need to consider familial inheritance even in the absence of a family history, emphasizing the importance of familial genetic screening and genetic counseling for early identification of potential cases. Such results further underscore the necessity of investigating the genetic characteristics of familial NIID and their implications for offspring. Genetic counseling is therefore essential for all affected families, and long-term clinical surveillance with regular neurological, cognitive and urological assessments is recommended for the minor carrying the NOTCH2NLC GGC repeat expansion, to enable early identification and intervention of potential disease manifestations and improve clinical outcomes.

Bladder dysfunction is a common manifestation of NIID, and prior reports have documented that urinary symptoms may precede neurological manifestations in some NIID patients. However, this case differs meaningfully from previous studies in four key aspects: first, the proband presented with isolated nocturnal overflow incontinence as the sole initial and dominant symptom for an 8-year long course, with no other neurological or systemic symptoms during this period, which is a rare and specific phenotypic feature not described in prior reports; second, this is the first reported familial NIID case with urinary symptoms preceding neurological features, in contrast to the sporadic cases reported previously; third, the case developed severe urological damage including severe neurogenic bladder, bilateral hydroureter and hydronephrosis with 375 ml residual urine, which is more severe than the mild to moderate urinary dysfunction noted in prior studies; fourth, conventional urological treatment and symptomatic drugs showed no significant efficacy, which supplements the clinical understanding of treatment resistance in NIID-related urinary dysfunction. In addition, the variable NOTCH2NLC GGC repeat expansion counts in family members and its correlation with the unique urinary phenotype provide new empirical data for the genotype–phenotype correlation of familial NIID. Bladder dysfunction, including frequency, urgency, incontinence, and retention, is a common clinical manifestation of NIID. Benign prostatic hyperplasia was excluded as the sole cause of the patient’s persistent severe urinary symptoms due to the lack of symptomatic improvement after transurethral prostatectomy, with neurogenic bladder secondary to NIID confirmed as the definitive etiology. A study of 223 NIID patients reported that 55.6% exhibited rectal and bladder dysfunction [18], with urinary symptoms preceding neurological manifestations by up to 12 years in some cases [19]. Yuan *et al.* similarly documented urinary symptoms (for example, frequency, weak stream, incontinence) in 11 of 15 NIID patients, with severe cases progressing to ureteral dilation, hydronephrosis, and even renal failure due to chronic retention [20]. Our case, presenting with overflow incontinence as the initial symptom, accompanied by severe urinary retention and ureteral dilation, aligns with these findings and reinforces the clinical significance of bladder dysfunction in NIID. The underlying mechanism of incontinence in NIID remains unclear but may involve the synergistic effects of central and peripheral factors. Central involvement may arise from NIID-related lesions in the cerebral cortex, basal ganglia, or brainstem (key regions regulating micturition), while peripheral involvement could stem from autonomic neuropathy affecting bladder-innervating nerves, leading to detrusor dysfunction or urethral sphincter imbalance. The coexistence of cognitive impairment and peripheral neuropathy in our patient supports this dual-mechanism hypothesis, though further research is warranted to elucidate the precise molecular pathways. This case identifies the unnecessary prostate surgery as a key clinical error from misattributing NIID-related neurogenic bladder symptoms to BPH, offering a constructive cross-disciplinary learning point: urologists should prioritize urodynamic assessment and rule out neurological etiologies for atypical lower urinary tract symptoms, while neurologists must recognize autonomic dysfunction like neurogenic bladder as a potential early presentation of neurodegenerative disorders such as NIID, emphasizing critical collaboration between the two specialties to avoid misdiagnosis and unnecessary interventions.

A large-scale Chinese multicenter study of 247 NIID patients reported the incidence of limb weakness and sensory disturbance as 22.0% and 14.8%, respectively [2]. Notably, 49.1% of cases exhibited subclinical peripheral neuropathy on neurophysiological testing, even in the absence of overt symptoms or signs [2]. Our case’s presentation of peripheral nerve injury is consistent with this finding, further confirming the high prevalence of peripheral neuropathy in NIID and highlighting the need for routine neurophysiological assessment to comprehensively evaluate disease burden.

NIID diagnosis and treatment remain challenging due to its marked clinical heterogeneity, particularly in patients presenting with autonomic dysfunction, who are prone to misdiagnosis or delayed diagnosis [21]. Brain magnetic resonance imaging (MRI) is a key diagnostic tool, with hyperintensities at the corticomedullary junction on diffusion-weighted imaging (DWI), known as the "lace sign" or "diaper sign," being a typical radiological hallmark [1]. However, these findings are not entirely specific, as some patients may only present with white matter lesions or atrophy, necessitating a comprehensive assessment combining clinical manifestations, imaging, and other tests [22]. Pathological examination (for example, detection of intranuclear eosinophilic inclusions in skin biopsies) is the gold standard for diagnosis, but genetic testing serves as a crucial alternative in resource-limited settings. GGC repeat expansion (> 60 repeats) in the NOTCH2NLC 5′-UTR is the primary genetic cause of NIID, and our patient was confirmed via PCR-capillary electrophoresis (115 repeats). Notably, initial whole-exome sequencing (WES) yielded negative results, indicating that targeted NOTCH2NLC testing is more sensitive and specific for NIID diagnosis—likely due to limited coverage of non-coding regions (for example, 5′-UTR) in WES. This confirms that WES may miss 5′-UTR repeat expansions, and targeted NOTCH2NLC testing should be prioritized for suspected NIID cases, especially those with atypical clinical or radiological features. In this case, the patient presented with urinary incontinence as the initial symptom and was initially misdiagnosed with benign prostatic hyperplasia (BPH) at an outside hospital, where no formal urodynamic study was performed preoperatively, no preoperative data were retained, and transurethral resection of the prostate (TURP) was performed merely based on the presumed diagnosis of BPH from urinary retention, lower urinary tract symptoms (LUTS), and prostatic enlargement; during the current admission to our hospital, the patient’s postoperative total prostate-specific antigen (tPSA) was measured at 2.640 ng/mL (free/tPSA 0.307), and imaging revealed residual prostatic enlargement (~ 5.0 cm transverse diameter) with calcification consistent with prior TURP, with the underlying NIID-related neurogenic bladder unrecognized at the outside hospital rendering the surgery an unnecessary intervention due to misdiagnosis and the patient’s urinary tract symptoms showing no significant improvement postoperatively, and the final diagnosis of NIID was confirmed in our hospital via imaging and genetic testing, underscoring the importance of considering NIID in elderly males with refractory urinary symptoms.

Regarding treatment, NIID currently lacks disease-modifying therapies, with management focusing on symptomatic support [23]. Conventional medications (for example, finasteride, tamsulosin) and acupuncture failed to alleviate our patient’s incontinence, reflecting the therapeutic challenges of NIID-related neurogenic bladder. Sacral neuromodulation (SNM) has shown efficacy in some neurogenic bladder patients by regulating sacral nerve reflex pathways [24], but its application in NIID remains rarely reported and controversial. Future studies should explore the feasibility of SNM in NIID patients based on individual neurophysiological profiles. Emerging therapies targeting NOTCH2NLC GGC expansion, such as RNA interference, antisense oligonucleotides, CRISPR-based strategies, and small-molecule RNA drugs [25], hold promise but require further preclinical and clinical validation to establish safety and efficacy. Elucidating the pathological mechanisms underlying GGC repeat expansion-induced neuronal damage is critical for developing targeted therapies to improve patient outcomes.

## Conclusion

This case highlights the remarkable clinical heterogeneity and diagnostic complexity of neuronal intranuclear inclusion disease (NIID). The patient initially presented with isolated urinary incontinence and later developed mild cognitive impairment, underscoring the need to consider NIID in patients with unexplained autonomic or cognitive symptoms. The identification of a NOTCH2NLC gene expansion confirmed a hereditary form of NIID, extending the known clinical and genetic spectrum of this disorder. These findings emphasize the diagnostic value of genetic testing and family screening in suspected NIID cases and provide important insights for early recognition and timely clinical intervention.

## Data Availability

The data that support the conclusions of this article are included within the article.

## Abbreviations

NIID: Neuronal intranuclear inclusion disease
MRI: Magnetic resonance imaging
DWI: Diffusion-weighted imaging
PCR: Polymerase chain reaction
5′ UTR: 5′ Untranslated region
CMAP: Compound muscle action potential
SNAP: Sensory nerve action potential
MCV: Motor conduction velocity
SCV: Sensory conduction velocity
BPH: Benign prostatic hyperplasia
